# Effectiveness of three oral hygiene regimens on oral malodor reduction: a randomized clinical trial

**DOI:** 10.1186/s13063-015-0549-9

**Published:** 2015-01-27

**Authors:** Ei Ei Aung, Masayuki Ueno, Takashi Zaitsu, Sayaka Furukawa, Yoko Kawaguchi

**Affiliations:** Department of Oral Health Promotion, Graduate School of Medical and Dental Sciences, Tokyo Medical and Dental University, 1-5-45 Yushima, Bunkyo-ku, Tokyo, 113-8549 Japan

**Keywords:** Chlorine dioxide, Mouth washing, Oral malodor, Tongue cleaning, Tooth brushing, Volatile sulfur compounds

## Abstract

**Background:**

Breath odor is a nuisance problem for many people around the world. Bad breath affects social interactions of people in daily life by causing personal discomfort and emotional stress. There are chemical and mechanical methods for controlling oral malodor. Many studies of various mouth rinse applications and tongue cleaning procedures have been conducted. However, few studies have compared the effect of simultaneous chemical and mechanical procedures on the reduction of volatile sulfur compounds (VSCs) in subjects with oral malodor. The purpose of this study was to assess the effects of different oral hygiene procedures on reduction of VSCs in subjects with oral malodor.

**Methods:**

Thirty male volunteers who matched with study criteria were divided randomly into two groups. Both groups performed tooth brushing, mouth washing with chlorine dioxide, tongue cleaning and combination of those in different sequence for five weeks. Total VSCs of subjects were measured with a Breathtron®, and oral health status was also examined. Quantitative analyses were performed using the Statistical Package for Social Science (SPSS 16.0).

**Results:**

There were no significant differences in oral health status between the two groups at the baseline. No significant decrease in oral malodor was detected after one week of tooth brushing. Significant reductions in VSCs were shown by adding mouthwash or tongue cleaning to tooth brushing from the second week to fourth week (*P* <0.01). The greatest reduction in VSCs was found at the fifth week after the practice of all three oral hygiene regimens.

**Conclusions:**

Tooth brushing alone does not significantly reduce oral malodor. Mouth washing and tongue cleaning significantly reduce oral malodor, but combining tooth brushing, mouth washing and tongue cleaning regimens is most effective for oral malodor reduction. The results of this study could contribute to the formulation of appropriate preventive strategies against oral malodor not only for the general public but also for dental professionals serving as oral malodor-related service providers.

**Trial registration:**

Registration number - ClinicalTrials.gov NCT02113137. Registration date – April 7th, 2014.

**Electronic supplementary material:**

The online version of this article (doi:10.1186/s13063-015-0549-9) contains supplementary material, which is available to authorized users.

## Background

Breath odor is a nuisance problem for many people around the world. Bad breath affects social interactions of people in daily life by causing personal discomfort and emotional stress. Previous studies have reported that about 30% to 50% of the population present with a problem of bad breath [1-3]. Breath odor evaluation should be performed carefully given that the degree of breath odor varies widely throughout the daily circadian rhythm. When the odor is beyond the level of social acceptance it is termed bad breath or oral malodor.

The main causative substances of oral malodor are volatile sulfur compounds (VSCs) produced by bacteria and protein putrefaction of sulfur-containing amino acids. There are various etiological factors for oral malodor, but intra-oral sources, such as periodontal diseases, tongue coating, poor oral hygiene, and dry mouth, are the main causes of increased levels of VSCs [4,5]. Because oral malodor arises from many causes, proper examination, diagnosis, and treatment are essential to improve the condition [6]. Any area in the oral cavity where microorganisms, plaque, and oral debris accumulate can produce VSCs [7,8]. The primary source of VSCs production is a coating on the dorsum of the tongue since the tongue forms a distinct environment for the accumulation of microorganisms, desquamated epithelium cells, and food debris [4,9].

Both chemical and mechanical methods are available for controlling oral malodor. For example, oral malodor can be diminished by reducing the amount of food debris or causative bacteria in the oral cavity [1,4,10] or by converting VSCs to non-volatile compounds [11]. Lay people try to ameliorate bad breath through various procedures, including limiting their meals, drinking high amounts of water, increasing the time spent brushing their teeth, and using anti-bacterial mouthwash or masking products.

Poor oral hygiene is not only closely linked to various oral health problems, but also has a significant effect on oral malodor. Mechanical tooth cleaning, such as tooth brushing or interdental flossing, is an essential daily oral hygiene practice, but many articles have revealed that tooth brushing alone will not significantly reduce oral malodor [12,13]. On the other hand, mouth rinsing and tongue cleaning can reduce VSCs levels [10].

Many types of mouthwash are sold in the market for oral malodor prevention and the effects of mouthwash on oral malodor by way of bactericidal, bacteriostatic, or oxidative action have been previously studied [11]. Chlorine dioxide (ClO_2_) mouthwash has a strong oxidative effect on amino acids, the precursors of VSCs. Previous research has indicated that the use of ClO_2_ mouthwash effectively reduces total VSCs in oral malodor patients [14,15]. Further, tongue cleaning is effective in preventing bacterial putrefaction on the tongue by reducing the amount of tongue coating. By decreasing the nutrient supplies to the bacteria, bacteria counts on the tongue and total VSCs in the oral cavity are reduced [16-18]. Many studies of various mouth rinse applications and tongue cleaning procedures have been conducted [19-21]. However, few studies have compared the effect of simultaneous chemical and mechanical procedures on the reduction of VSCs in subjects with oral malodor. Therefore, this study aims to assess the effects of different oral hygiene procedures, i.e., tooth brushing, mouth washing, and tongue cleaning, alone and in combination, on the reduction of VSCs in subjects with oral malodor.

## Methods

### Subjects

Sample size was determined with an expected mean VSCs (parts per billion; ppb) difference of 50, a standard deviation of 60, a 95% confident interval, and a power of 80% from a pilot previously study conducted. The results indicated that 12 subjects in each group were required for the study and 15 in each group would be safe when considering subject dropouts.

This clinical study was conducted in Yangon, Myanmar, from September to October of 2013. Forty-eight male monk volunteers were screened to assess whether they matched the inclusion criteria, which included no systemic diseases, no current use of antibiotics, no severe dental caries, no periodontal pocket more than 3 mm in depth, no history of allergy to any kind of mouthwash, no habits of smoking or betel quid chewing, and total VSCs more than the threshold level of 250 ppb measured by Breathtron® (Yoshida, Tokyo, Japan). Eighteen of these subjects were excluded because 13 had breath odor below 250 ppb and 5 had betel quid chewing habits (Additional file 1). After the screening, the final subjects used for this study were 30 males aged 18 to 30 years (mean age: 20.18 ± 2.8 years). Prior to the study, the study protocol was explained to the subjects and they all signed a consent form for participation.

### Study design and procedures

**Figure 1:**
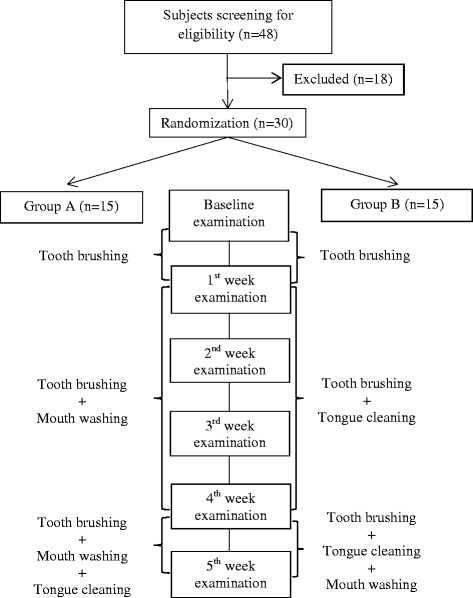
**Diagram of study protocol.**

This study had a randomized, single blind, 5-week parallel design (Figure 1). Subjects were randomly divided into two groups (A and B) of 15 subjects each by a computer-generated randomization system. Subjects were allocated to each group using random sequences by a person not related with the current study (Additional file 2). Both groups were instructed to brush their teeth with a scrubbing method by using their own toothbrush in the first week to assess the effect of tooth brushing on oral malodor. Toothpaste usage depended on the subject’s choice. For the next 3 weeks, both groups continued tooth brushing; group A used 12 mL of chlorine dioxide (ClO_2_) Fresh® mouthwash (Bio-Cide International, Inc., Oklahoma, USA and Pine Medical Co., Tokyo, Japan) for 30 seconds twice daily, and group B performed tongue cleaning twice daily with a small toothbrush. The subjects were instructed to use mouthwash or perform tongue cleaning after waking up in the morning (between 7:00 am and 9:00 am) and before going bed at night (between 9:00 pm and 11:00 pm).

For tongue cleaning, we provided the same small toothbrush to all the subjects and they were instructed in accordance with the following standardized procedure. The small toothbrush is wet with clean water before tongue cleaning. Place the tongue forward as far as possible and place the toothbrush on the posterior part of the tongue. Move the toothbrush forward slowly and steadily and then clean the toothbrush with running water to remove debris. Repeat the above procedure until no debris is attached to the toothbrush and confirm the cleanness of the tongue with a mirror.

Following the above, both groups practiced all three oral hygiene regimens: tooth brushing, mouth washing, and tongue cleaning, for the next week. Total VSCs, debris index (DI) score, bleeding on probing (BOP), and tongue coating were examined at the baseline and weekly during the 5 weeks by a principal investigator who was blinded to the examined subject’s group.

### Measurements

#### Oral malodor evaluation

Oral malodor was evaluated during the day from 2 pm to 5 pm. Subjects were asked to refrain from drinking and eating as well as oral hygiene practices at least 2 hours before the measurement. The amount of total VSCs was measured using a portable sulfide monitoring device (Breathtron®) [22,23]. Subjects were requested to close their mouth tightly for 3 minutes before the measurement. A disposable mouthpiece was inserted into the subject’s mouth for 45 seconds while they closed their mouth tightly and breathed through their nose. The amount of total VSCs was shown in the display in units of ppb. Subjects with a level of total VSCs of more than 250 ppb were categorized as having oral malodor.

#### Oral health status

Following the oral malodor evaluation, dentition status [number of decayed teeth (DT), number of filled teeth (FT), and number of missing teeth (MT) excluding third molars] was examined. The amount of plaque was evaluated with the debris index (DI) of the Oral Hygiene Index (OHI) [24]: 0 = no debris or stain present; 1 = soft debris covering not more than one third of the tooth surface being examined, or the presence of extrinsic stains without debris, regardless of surface area covered; 2 = soft debris covering more than one third but not more than two thirds of the exposed tooth surface; 3 = soft debris covering more than two thirds of the exposed tooth surface. The highest score for each tooth was recorded. Four sites (mesial, buccal, distal, and lingual) of all teeth were examined for BOP. Gingival bleeding was recorded if bleeding was detected after examination with a periodontal probe (University of North Carolina, UNC-15).

#### Tongue coating

Tongue coating was evaluated by a modified Winkel tongue-coating index [25]. The tongue dorsum was divided into nine areas and tongue coating was evaluated for all nine areas with a score of 0 = no coating, 1 = a light coating (a thin tongue coating with clearly visible papillae), and 2 = a thick coating (a dense coating totally covered the papillae and they were not visible) [15]. The tongue coating score was calculated by adding the scores of all nine areas, resulting in a possible range from 0 to 18.

#### Saliva measurement

Subjects were requested to spit out all saliva into a collecting paper cup for 5 minutes. The flow rate of saliva (mL/min) was calculated, and the saliva pH level was measured with a bromothymol blue test paper.

### Ethical approval

The Ethical Committee for Human Research at Tokyo Medical and Dental University approved this clinical study (Approval No.850) and the study protocol was also approved by the University of Dental Medicine (Yangon) in Myanmar. The trial was registered with Clinical-Trials.gov protocol registration system, NCT02113137.

### Data analysis

Statistical analysis was performed using the Statistical Package for Social Science (SPSS 16.0). The independent sample *t*-test was used to determine significant differences of means between the two groups with the significance level set at *P* <0.05. The one-way repeated-measure ANOVA test was applied for the mean changes of variables between the baseline and the following weekly examinations.

## Results

### Baseline characteristics of the subjects

Table 1 shows baseline characteristics of the subjects in groups A and B. There were no significant differences in any baseline characteristics, including age, total VSCs, present teeth (DT, FT, MT), flow rate and pH of saliva, DI, BOP, and tongue coating between groups A and B.

**Table 1:** **Baseline characteristics of the subjects**

**Variables**	**Group A (n = 15)**		**Group B (n = 15)**		***P*** value*
	**Mean**	**± SD**	**Mean**	**± SD**	
Age	19.80	2.90	21.10	3.50	0.27
Volatile sulfur compounds (ppb)	345.5	87.5	468.7	244.4	0.08
Present teeth	27.60	0.51	27.87	0.35	0.11
Decayed teeth	0.13	0.35	0.00	0.00	0.16
Filled teeth	0.07	0.26	0.00	0.00	0.33
Missing teeth	0.40	0.51	0.13	0.35	0.11
Saliva flow rate	0.58	0.19	0.48	0.17	0.14
Saliva pH	7.00	0.39	7.05	0.40	0.75
Debris index	0.83	0.20	0.89	0.24	0.47
Bleeding on probing	12.47	7.62	12.07	8.97	0.90
Tongue coating	12.40	4.19	11.47	5.21	0.59

*Comparison between group A and group B by independent sample *t*-test.

### Changes of oral health status

Table 2 shows the changes of mean values of DI, BOP, and tongue coating. Compared to the baseline, DI scores significantly improved in both groups at the first week’s examination (*P* <0.01) and low scores were maintained at the following weekly examinations. There was no significant difference in DI scores between groups A and B at any examination period.

**Table 2:** **Changes of DI, BOP, and tongue coating**

**Variables**	**Group**	**Baseline**		**1st week**		**2nd week**		**3rd week**		**4th week**		**5th week**	
		**Mean**	**± SD**	**Mean**	**± SD**	**Mean**	**± SD**	**Mean**	**± SD**	**Mean**	**± SD**	**Mean**	**± SD**
Debris index	A	0.83	0.20	0.18	0.15	0.12	0.20	0.15	0.15	0.24	0.20	0.15	0.16
	B	0.89	0.24	0.26	0.17	0.14	0.12	0.21	0.14	0.27	0.20	0.13	0.12
	*P* value*	0.470		0.200		0.630		0.310		0.740		0.700	
Bleeding on probing	A	12.47	7.62	2.07	5.11	1.47	4.22	1.53	4.67	0.53	1.60	0.80	3.09
	B	12.07	8.97	4.20	4.92	0.87	2.23	0.47	0.64	0.47	1.30	0.27	0.70
	*P* value*	0.900		0.250		0.630		0.390		0.900		0.520	
Tongue coating	A	12.40	4.19	9.00	2.95	4.33	4.44	5.71	5.36	5.60	4.41	0.20	0.56
	B	11.47	5.21	11.87	4.63	0.87	2.30	2.33	3.77	0.92	1.80	0.27	0.79
	*P* value*	0.590		0.054		0.014		0.058		0.001		0.790	

*Comparison between group A and group B by independent sample *t*-test.

In comparison with the baseline, BOP significantly decreased at the first week’s examination in both groups (*P* <0.05). At the following weekly examinations, BOP maintained low values in both groups. There was no significant difference in BOP between groups A and B at any examination period.

Compared with the baseline, tongue coating score did not change significantly at the first week’s examination in either group, but it significantly decreased from the second week’s examination in both groups (*P* <0.05). Group B had significantly lower tongue coating scores than group A at the second and fourth weeks. At the third week, group B had lower tongue coating score than group A but there was no significant difference between the two groups. At the fifth week, the tongue coating score in both groups was at its lowest, and there was no significant difference in the tongue coating score between groups A and B.

### Changes of oral malodor

**Figure 2:**
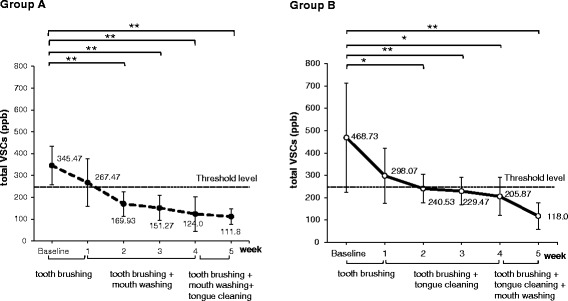
**Changes of mean total VSCs (ppb) in groups A and B.**

Figure 2 shows the changes of oral malodor in each group. At the first week’s examination, there were no significant reductions of total VSCs compared to baseline in either group. In both groups, the mean value of total VSCs level was above 250 ppb, and there was no significant difference in the total VSCs between the two groups.

At the second week, total VSCs significantly decreased from baseline in both group A (*P* <0.01) and group B (*P* <0.05). However, the smaller *P* value in group A showed that group A had a higher confidence level than group B. From the second to the fourth week, both groups had significant reductions of total VSCs compared to baseline, and all mean values were lower than 250 ppb. Group A showed significantly lower total VSCs values than group B.

At the fifth week, the total VSCs were significantly lower than at baseline and lower than the previous weekly examinations in both groups. The total VSCs in each group decreased to 111.8 ppb in group A and 118.0 ppb in group B, and there was no significant difference in the total VSCs between the two groups.

**Figure 3:**
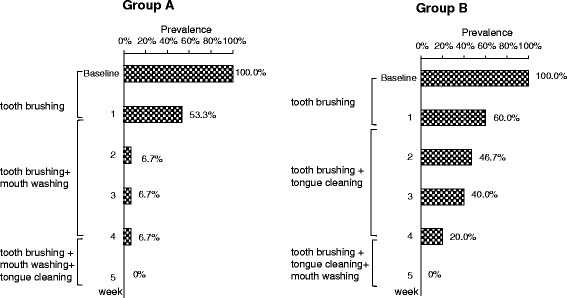
**Changes of the percentage of subjects with oral malodor in groups A and B.**

The change of the percentage of the subjects with oral malodor is shown in Figure 3. At the first weekly examination, more than 50% of the subjects in both groups still had oral malodor. The percentage of subjects with oral malodor in group A decreased to 6.7% on the second week, and that in group B declined to 20.0% by the fourth week. At the fifth week, no subjects in either group had oral malodor.

## Discussion

The current study, which examined the effect of chemical and mechanical procedures on reducing oral malodor for up to 5 weeks, revealed that tooth brushing alone could not improve the oral malodor, but either mouth washing or tongue cleaning significantly reduced the VSCs. Further, the combination of tooth brushing, mouth washing and tongue cleaning was the most effective regimen for improvement of oral malodor.

Few studies have examined the combined effects of chemical and mechanical methods on oral malodor. Moreover, most studies evaluated only transient effects of the procedures, although a very few studies investigated long-term (i.e., more than 3 weeks) effects [26-28]. For an oral malodor study, selection of subjects is crucial because many different factors affect oral malodor. To exclude the influence of oral and systemic diseases on oral malodor, healthy young male adults were recruited for this study. Because all subjects were male, the menstrual cycle, which can affect oral malodor, was not a factor [29]. Further, all subjects, as full time monastery residents, lived in the same place and had a similar life style, including the content, time, and frequency of meals. Thus, the problem of different food and eating habits, which could affect oral malodor [30], were avoided in the present study.

Oral malodor was examined with the Breathtron® sulfide monitoring instrument in the current study. Previous studies reported a significant association between the organoleptic test (the gold standard for clinical oral malodor diagnosis) and sulfide monitoring measurements [22,23]. The Breathtron® is not only a reliable machine for oral malodor measurement but also is suitable for field or clinical study because of its portability, simplicity, and speed.

In this study, 30 recruited subjects out of 48 (63%) had oral malodor. Since the sample population included only monks, the prevalence of 63% could not be generalized to the whole Myanmar population. As Table 1 shows, the number of teeth with BOP and tongue coating scores were high at the baseline. Therefore, we assume that poor oral hygiene may be one of the reasons for higher prevalence of oral malodor. We employed a scrubbing method for tooth brushing because it is one of the most commonly used brushing methods to reduce plaque and prevent periodontal disease [31]. Although oral health conditions, such as dental plaque and BOP, improved, tooth brushing alone could not reduce VSCs significantly. This is probably because one of the main causes of oral malodor in periodontally-healthy subjects is not plaque but rather a tongue coating. A previous study also demonstrated that oral malodor of subjects in a tooth-brushing group was more severe than those in a tongue-cleaning plus tooth-brushing group [13].

Various kinds of mouthwashes that contain chemicals, such as chlorhexidine, zinc, triclosan, ClO_2_, and cetylpyridinium chloride are available on the market [32-34]. Shinada et al. [14,15] demonstrated that a ClO_2_ mouthwash could reduce total VSCs level. In our study, a 12 mL single-use disposable pack of ClO_2_ was used for the mouth wash. The present results showed that this ClO_2_ mouthwash reduced VSCs significantly and kept the VSCs level low during the study period (as long as 4 weeks in this study). ClO_2_ has a powerful oxidative action to change VSCs to non-malodorous products, and the chlorite anion exerts bactericidal activity against oral malodor-producing microorganisms. Further, our study found that the amount of tongue coating was significantly reduced when using a ClO_2_ mouthwash without tongue cleaning. Previous studies have also demonstrated that a mouthwash could reduce the bacterial count on the dorsum of the tongue and the amount of tongue coating [35]. Some mouthwashes have side effects such as a burning sensation, staining, or taste problems [25,34]. A previous study using ClO_2_ mouthwash reported no measurable side effects in the oral cavity [14]. Similarly, in this study, no one complained about oral mucosa irritation, discoloration, or taste changes after using the mouthwash.

Tongue coating plays a vital role in the production of VSCs, not only in patients without periodontal diseases but also in periodontitis patients. Microorganisms from a tongue coating potentially contribute dental plaque accumulation and periodontal disease progression [36]. Subjects were instructed to clean their tongue using a small toothbrush. Although a tongue brush is preferable for tongue cleaning, tongue brushes are not common in Myanmar and are difficult to obtain. Moreover, previous studies indicated that regular practice was important for effective tongue cleaning [13]. Therefore, we used small toothbrushes for tongue cleaning in this study.

Adding tongue cleaning to tooth brushing significantly reduced the VSCs as well as the percentage subjects with oral malodor by up to 80% in this study. It is reported that tongue cleaning is two times more effective than tooth brushing for oral malodor reduction [13,27]. Tongue cleaning decreases the concentration of VSCs by disturbing the formation of a tongue biofilm and by reducing the debris and bacterial load in the oral cavity [9,37]. Seemann et al. [38] reported that regular practice of tongue cleaning procedure had a long lasting effect on the reduction of oral malodor.

Comparison of oral malodor reduction between groups A and B showed that the percentage of subjects with oral malodor quickly declined to 6.7% at the second week’s examination in group A. On the other hand, the decrease was more gradual in group B, from 46.7% at the second week to 20% at the fourth week. It was probably because the actions of mouthwash, namely its antimicrobial properties and oxidization of VSCs to non-malodorous products, had a prompt effect on oral malodor improvement in group A. The subjects in group B, who at first used tongue cleaning, took time to learn and increase the skills required for complete cleaning of the tongue. This suggests that mouthwash may be easier to use and give more rapid results, while tongue cleaning needs skill and time to become fully effective, especially for people who are introduced to the regimen for the first time.

This study demonstrates that a combination of chemical and mechanical procedures has the strongest effect on the reduction of VSCs. One former study reported that using mouthwash was essential for additional reduction of oral malodor if the subject still had oral malodor after tooth and tongue brushing [36]. Our findings agree with that result, in that mechanical tongue cleaning without using any kind of chemical agents could reduce the VSCs but was not more effective than combining methods. Many studies have demonstrated that a combination of tongue cleaning and chemical products, such as mouthwash and dentifrices, are more efficacious than any single method for oral malodor reduction [11,27,39]. This suggests that the chemical action of a mouthwash can help reduce oral malodor by reaching areas that are difficult to access by tongue cleaning, especially the posterior one-third of the tongue [9,26].

One limitation of this study was that we did not control the kind or usage of toothpaste, which might have affected oral health status as well as oral malodor. However, we considered the influence of toothpaste to be negligible because the randomization of the subjects yielded no significant differences in baseline oral health status or oral malodor between the two groups.

Previous epidemiological studies have reported that oral malodor is a widespread problem in many countries; therefore, it should receive further attention from medical and dental specialists. Moreover, lay people do not know exactly which oral hygiene practices in their daily life will prevent oral malodor and maintain fresh oral breath. The results of this study could contribute to the formulation of appropriate preventive strategies against oral malodor not only for the general public but also for dental professionals serving as oral malodor-related service providers. However, we recommend further study to examine the longer-term effect of mouthwash and tongue brushing on oral malodor, and to assess oral health conditions, including changes of oral microorganisms, in the continuous practice of these procedures.

## Conclusions

The results of this study indicate that both mouth washing, as a chemical method, and tongue cleaning, as a mechanical method, significantly reduce oral malodor. However, combining both mechanical and chemical regimens is the most effective method for the reduction of VSCs in subjects with oral malodor.

## Additional files

Additional file 1:
**CONSORT flow diagram.**
Additional file 2:
**CONSORT checklist.**

## Abbreviations

ClO_2_: Chlorine dioxide
BOP: Bleeding on probing
DI: Debris index
DT: Decayed teeth
FT: Filled teeth
MT: Missing teeth
OHI: Oral hygiene index
VSCs: Volatile sulfur compounds
