# Supplementary material for: Effectiveness of three oral hygiene regimens on oral malodor reduction: a randomized clinical trial
Source: Trials. 2015 Jan 27;16:31. doi: 10.1186/s13063-015-0549-9 (PMC4324034; doi:10.1186/s13063-015-0549-9)
Supplement: Additional file 1: — CONSORT flow diagram. [file 13063_2015_549_MOESM1_ESM.doc]

**
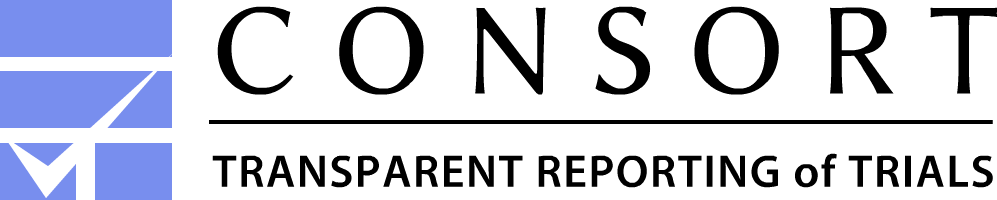
**

**CONSORT 2010 Flow Diagram**

**Allocation**

**Analysis**

**Follow-Up**

**Enrollment**

Assessed for eligibility (n=48)

Excluded (n=18)

♦  Not meeting inclusion criteria (n=13)

♦  Declined to participate (n=0 )

♦  Other reasons (n=5)

Analysed (n=15)
♦ Excluded from analysis (give reasons) (n=0)

Lost to follow-up (give reasons) (n=0)

Discontinued intervention (give reasons) (n=0)

Allocated to intervention (n=15)

♦ Received allocated intervention (n=15)

♦ Did not receive allocated intervention (give reasons) (n=0)

Lost to follow-up (give reasons) (n=0)

Discontinued intervention (give reasons) (n=0)

Allocated to intervention (n=15)

♦ Received allocated intervention (n=15)

♦ Did not receive allocated intervention (give reasons) (n=0)

Analysed (n=15)
♦ Excluded from analysis (give reasons) (n=0)

Randomized (n=30)
